# Robotic management of superior mesenteric artery syndrome after weight loss: a case report

**DOI:** 10.1093/jscr/rjag471

**Published:** 2026-06-15

**Authors:** Cristina Kontogiannis, Sami Asif, Amit Kharod

**Affiliations:** College of Osteopathic Medicine, University of New England, 11 Hills Beach Road, Biddeford, ME 04005, United States; Rowan-Virtua School of Osteopathic Mediciney, 42 E. Laurel Rd, Stratford, NJ 08084, United States; Department of Surgery, Centrastate Medical Center, 901 West Main Street, Freehold, NJ 07728, United States

**Keywords:** superior mesenteric artery syndrome, duodenojejunostomy, robotic surgery, intestinal obstruction, weight loss

## Abstract

Superior mesenteric artery (SMA) syndrome is a rare cause of proximal intestinal obstruction resulting from compression of the third portion of the duodenum between the aorta and the SMA. It most commonly occurs in slender individuals or those experiencing rapid weight loss, but presentations can be atypical and diagnostically challenging. We report the case of a 28-year-old female with history of postural orthostatic tachycardia syndrome and gastroparesis who presented with recurrent nausea and intractable vomiting following a 110-lb weight loss after prior obesity. Upper gastrointestinal endoscopy and gallbladder ultrasonography were unremarkable. Computed tomography of the abdomen demonstrated findings consistent with SMA syndrome, including severe narrowing of the aortomesenteric angle and reduced aortomesenteric distance. The patient subsequently underwent robotic-assisted duodenojejunostomy with resolution of symptoms. This case highlights an atypical presentation of SMA syndrome following massive weight loss and demonstrates the feasibility of robotic duodenojejunostomy as an effective treatment option.

## Introduction

Superior mesenteric artery syndrome is an uncommon and frequently underrecognized cause of proximal small-bowel obstruction resulting from external compression of the third portion of the duodenum between the SMA and the aorta [1]. The reported prevalence ranges from 0.013% to 0.3% [2]. Diagnosis is often challenging due to nonspecific symptoms such as nausea, vomiting, abdominal pain, and weight loss that overlap with more common gastrointestinal disorders [1].

Classic risk factors include a slender body habitus, rapid linear growth in adolescents, and severe cachexia associated with chronic illness [1, 3]. Despite being described more than a century ago, SMA syndrome remains a diagnosis that is frequently delayed until cross-sectional imaging demonstrates a narrowed aortomesenteric angle or decreased aortomesenteric distance [4].

Management strategies range from conservative measures such as nutritional rehabilitation to operative bypass procedures when conservative therapy fails [5]. Duodenojejunostomy is considered the surgical gold standard in patients with persistent symptoms [2, 6].

We present a case of SMA syndrome occurring after massive weight loss in a patient with prior obesity, complicated by prolonged, intractable emesis and malnutrition that was successfully treated with robotic duodenojejunostomy. This case highlights an atypical risk profile and illustrates the role of robotic minimally invasive surgery in the management of this rare condition.

## Case presentation

A 28-year-old female with a past medical history of postural orthostatic tachycardia syndrome, gastroparesis, and post-traumatic stress disorder presented to the emergency department with abdominal pain and intractable vomiting. The patient reported a prior motor vehicle collision in 2018 requiring sternotomy for associated injuries. Following this event, her weight increased from 150 lbs to 290 lbs. Over the following year she experienced a 110-lb weight loss, reaching a weight of 180 lbs at presentation.

During the two and a half years preceding admission, the patient reported persistent nausea and multiple daily episodes of bilious, non-bloody vomiting associated with poor oral intake. On the day of presentation, she developed worsening nausea and vomiting with inability to tolerate liquids, prompting evaluation in the emergency department and subsequent admission.

During hospitalization, a gastric emptying study demonstrated delayed gastric emptying consistent with gastroparesis. Upper gastrointestinal endoscopy was unremarkable. Right upper quadrant ultrasonography demonstrated gallbladder sludge without gallstones, pericholecystic fluid, or gallbladder wall thickening. Due to elevated liver function tests, a CCK-HIDA scan was performed and demonstrated no evidence of acute cholecystitis. A CT scan of the abdomen and pelvis demonstrated findings consistent with SMA syndrome, including severe narrowing of the aortomesenteric angle (15°) and a markedly reduced aortomesenteric distance of 2–3 mm. Focal narrowing of the third portion of the duodenum as it passed between the SMA and aorta was observed.

Given the patient’s symptoms and imaging findings, she underwent robotic-assisted duodenojejunostomy with lysis of adhesions: pneumoperitoneum was established via an optically guided trocar placed in the left upper quadrant, followed by placement of an 8-mm trocar in the right mid-abdomen. The omentum was reflected to expose the ligament of Treitz and the third portion of the duodenum. After docking of the robotic system, careful dissection was performed to mobilize the duodenum and expose the region of compression. The ligament of Treitz was dissected and the jejunum mobilized.

A jejunal segment distal to the duodenojejunal flexure was divided using a stapling device, and mesenteric division was completed with a vessel sealer. A side-to-side duodenojejunostomy was created between the third portion of the duodenum and the jejunum using a 45-mm stapler after enterotomies were created in each segment. The common enterotomy was closed in two layers using running barbed sutures.

The patient experienced mild postoperative nausea and vomiting that gradually resolved during recovery. At postoperative follow-up, she reported complete resolution of symptoms and was tolerating oral intake without difficulty.

## Discussion

Superior mesenteric artery syndrome is a rare and frequently overlooked cause of duodenal obstruction. It most commonly affects slender adolescents or individuals experiencing rapid weight loss due to chronic illness or severe malnutrition [1, 3].

The case presented here is unusual because the patient developed SMA syndrome following significant weight loss after a period of prior obesity. While rapid weight loss is a recognized risk factor, cases occurring after large post-obesity weight reduction remain uncommon and may represent an underrecognized risk population. Unlike previously reported cases of SMA syndrome, this case is further distinguished by the coexistence of gastroparesis, which significantly confounded the clinical presentation and contributed to a prolonged diagnostic course.

The patient’s prolonged history of nausea and vomiting highlights the diagnostic difficulty associated with SMA syndrome. Because symptoms are nonspecific, patients often undergo extensive evaluation before diagnosis is established. In this case, multiple diagnostic studies—including endoscopy and hepatobiliary imaging—were unrevealing before CT imaging ultimately demonstrated the characteristic narrowing of the aortomesenteric angle and distance. These findings underscore the importance of cross-sectional imaging when SMA syndrome is suspected.

The coexistence of gastroparesis in this patient added significant diagnostic complexity. Symptoms of delayed gastric emptying—including nausea, early satiety, and vomiting—closely overlap with those of SMA syndrome, increasing the risk of misattribution and delayed diagnosis. In this case, the presence of a confirmed gastric emptying delay may have initially reinforced a functional diagnosis, potentially obscuring the contribution of a mechanical obstruction. This overlap underscores the importance of maintaining a high index of suspicion for structural pathology in patients with persistent or refractory symptoms, even in the presence of a known motility disorder. Recognition of SMA syndrome in this context was critical in guiding appropriate surgical intervention and achieving symptom resolution.

Initial management typically involves conservative therapy aimed at nutritional rehabilitation to restore mesenteric fat and increase the aortomesenteric angle [5]. However, conservative management fails in a significant proportion of patients, and surgical intervention is required in up to 70% of cases [5].

Laparoscopic duodenojejunostomy is the preferred surgical approach due to its high success rate and reduced morbidity compared with open surgery [6]. Robotic duodenojejunostomy remains less frequently described but may offer technical advantages, including enhanced visualization and improved instrument dexterity during intracorporeal anastomosis. In this case, the robotic platform facilitated precise dissection around the ligament of Treitz and allowed for meticulous intracorporeal anastomosis, which may be particularly advantageous in patients with prior abdominal surgery or complex anatomy. These technical benefits contributed to a favorable outcome with complete symptom resolution.

This case highlights an important diagnostic and surgical learning point: SMA syndrome should be considered in patients with persistent vomiting and significant weight loss, even when alternative diagnoses such as gastroparesis are present, and early surgical intervention may be warranted when conservative management fails.

## Conclusion

SMA syndrome is a rare but important cause of proximal intestinal obstruction that can be difficult to diagnose due to nonspecific symptoms. This case illustrates an atypical presentation occurring after massive weight loss in a patient with prior obesity.

Clinicians should maintain a high index of suspicion for SMA syndrome in patients with persistent vomiting and significant weight loss, even in the presence of a known motility disorder. Early consideration of cross-sectional imaging is essential to avoid delays in diagnosis.

Robotic duodenojejunostomy provided safe and effective definitive treatment with complete symptom resolution. As robotic surgery continues to expand in gastrointestinal practice, it may represent a valuable minimally invasive option for the surgical management of SMA syndrome.
